# Efficacy of Non-invasive Ventilation Compared to Intubation in Pediatric Acute Respiratory Failure: A Meta-Analysis

**DOI:** 10.7759/cureus.105202

**Published:** 2026-03-14

**Authors:** Mahrukh Sajid, Bushra Mushtaq, Uzma Abdul Jabbar, Rohma Momna, Joham Anees, Arzoo Siddiqi, Malahat Sohaib, Fareeha F Khan, Syed Talha Bin Aqeel, Ali Zaidi

**Affiliations:** 1 Medicine, Liaquat College of Medicine and Dentistry, Karachi, PAK; 2 Medicine, Chiniot General Hospital, Karachi, PAK; 3 Medicine, Bahria University Medical and Dental College, Karachi, PAK; 4 Medicine, Jinnah Sindh Medical University, Karachi, PAK; 5 Medicine, Karachi Medical and Dental College, Karachi, PAK; 6 Medicine, Dow University of Health Sciences, Karachi, PAK; 7 Medicine, Liaquat National Hospital and Medical College, Karachi, PAK; 8 Medicine, The Kidney Center, Karachi, PAK; 9 Medicine, United Medical and Dental College, Karachi, PAK; 10 Internal Medicine, Abbasi Shaheed Hospital, Karachi, PAK

**Keywords:** bilevel positive airway pressure, bipap, continuous positive airway pressure, cpap, intubation, meta-analysis, non-invasive ventilation, pediatric acute respiratory failure, respiratory support

## Abstract

Acute respiratory failure (ARF) in children is a major cause of morbidity and Pediatric Intensive Care Unit (PICU) admission and often requires timely respiratory support to prevent deterioration. Traditionally, invasive mechanical ventilation has been the cornerstone of management, yet it carries risks such as ventilator-associated pneumonia, airway injury, and sedation-related complications. Non-invasive ventilation (NIV), including Continuous Positive Airway Pressure (CPAP) and Bilevel Positive Airway Pressure (BiPAP), has emerged as a promising alternative that may reduce the need for intubation while maintaining adequate ventilation. However, evidence comparing the efficacy of NIV with intubation in pediatric ARF remains fragmented across diverse study designs and clinical contexts. This meta-analysis aimed to evaluate the comparative efficacy and safety of NIV versus invasive mechanical ventilation in pediatric patients with acute respiratory failure. Key clinical outcomes assessed included intubation rates, mortality, PICU length of stay, treatment failure, and ventilation-related complications. A comprehensive literature search was conducted across PubMed, Embase, Cochrane Library, Web of Science, and Google Scholar, supplemented by clinical trial registries and grey literature. Studies were included if they involved children under 18 years with ARF and compared NIV (CPAP/BiPAP) with intubation or standard therapy. Randomized controlled trials, prospective and retrospective cohorts, before-after studies, and meta-analyses were eligible. Data extraction and risk-of-bias assessments were performed independently by two reviewers using Cochrane Risk of Bias 2 tool (RoB 2) and Risk of Bias in Non-randomized Studies of Interventions (ROBINS-I) tools. Meta-analysis was conducted using random-effects models, and outcomes were summarized as risk ratios or mean differences where appropriate. Fifteen studies involving over 10,000 pediatric patients met the inclusion criteria. Across randomized trials and observational cohorts, NIV significantly reduced the need for intubation, with pooled estimates demonstrating a 21% relative reduction in intubation risk (risk ratio or RR=0.79, 95% CI 0.63-1.00). Early NIV failure commonly occurred within 24 hours and was associated with severe hypoxemia, pneumonia, and apnea. Mortality rates were low across all groups and did not significantly differ between NIV and invasive ventilation. Successful NIV use was associated with fewer invasive procedures, reduced ventilator-associated complications, and a trend toward shorter PICU stays, although hospital length of stay findings were variable across studies. NIV was generally well tolerated, with minimal complications primarily related to mask interface issues. This meta-analysis demonstrates that NIV is an effective and safe first-line therapy for pediatric ARF, substantially reducing the need for intubation without increasing mortality or complications. Its success is strongly dependent on early initiation, careful patient selection, and close monitoring within experienced PICU settings. While NIV does not appear to change survival outcomes, it meaningfully decreases procedure-related risks and supports more efficient use of critical care resources. Standardized protocols and further large-scale randomized trials are needed to refine indications, optimize modality selection, and strengthen evidence regarding long-term outcomes.

## Introduction and background

Clinical background

Acute respiratory failure (ARF) is a common, serious disease in pediatric intensive care units, and it refers to the inability of the respiratory system to maintain sufficient gas exchange. This may appear as hypoxemia (low blood oxygen levels), hypercapnia (elevated carbon dioxide levels in the blood), or a mixture of both, and it is capable of causing serious morbidity if not treated promptly. Pediatric ARF etiologies are numerous and varied, such as viral infections (respiratory syncytial virus: RSV), bacterial pneumonia, bronchiolitis, or acute crises of chronic respiratory diseases such as asthma and cystic fibrosis [1]. These are leading causes of pediatric intensive care unit [2] admissions globally and necessitate an immediate need for ventilatory support in order to avoid clinical deterioration.

For example, a child admitted with severe bronchiolitis who develops worsening respiratory distress may traditionally require intubation; however, early initiation of non-invasive ventilation may stabilize breathing and potentially avoid invasive ventilation. Despite improvements in supportive care, the treatment of pediatric ARF is difficult due to the variety of presentations of the disease, underlying pathophysiology and patient response and resilience towards therapy [3].

Current treatment approaches

Endotracheal intubation (placement of a breathing tube into the airway) and invasive mechanical ventilation (IMV) are established techniques of respiratory support in critically ill children. Although effective in establishing airway patency and controlling ventilation, intubation is not without significant risks, including ventilator-associated pneumonia, injury to the airway, sedation-related complications and prolonged PICU stay.

In recent years, the use of normal ventilation support has been replaced by non-invasive ventilation (NIV), which provides positive pressure assistance (delivery of pressurized airflow through a mask without inserting a breathing tube) and does not require an artificial airway [4]. NIV techniques, including continuous positive airway pressure (CPAP) and bilevel positive airway pressure (BiPAP), are being utilized more frequently in the pediatric intensive care unit. A possible benefit of NIV is that such expedited intubation can be avoided and exposure to invasive procedures minimized, while maintaining satisfactory oxygenation and ventilation to prevent complications related to these procedures.

Evidence gaps in current literature

Despite the wider use of NIV in the pediatric population, the benefit of NIV over IMV is still open to debate. A few studies have found that NIV decreases intubation rates and duration of PICU stay, while others indicate high failure rates for which patients had to be escalated towards invasive ventilation [5]. Adult NIV guidelines are referenced for general ventilatory principles whereas pediatric-specific guidance is limited. Mortality rates are low in children with ARF and inconsistently reported between studies. The primary therapeutic benefit of NIV may be avoiding invasive ventilation rather than reducing mortality.

Recent systematic reviews and network meta-analyses have investigated non-invasive respiratory support in children, but multiple studies report on NIV versus standard oxygen and high-flow nasal cannula (HFNC) rather than directly comparing it to IMV. In addition, the studies were heterogeneous in design, patient cohorts, and outcome definitions making it difficult to draw clear conclusions from the evidence. These gaps underscore the necessity of a well-directed synthesis that combines direct and indirect evidence, emphasizing clinical significance in the outcomes [6].

In addition, recent pediatric acute respiratory distress syndrome (ARDS) consensus statements, such as the Pediatric Acute Lung Injury Consensus Conference (PALICC) guidelines [7], emphasize individualized respiratory support strategies and careful selection of ventilatory modalities in children. Contemporary pediatric critical care guidelines and recent bronchiolitis trials evaluating non-invasive respiratory support further highlight the evolving role of NIV within stepwise respiratory management pathways. However, these sources primarily focus on broader respiratory support strategies rather than direct comparisons between NIV and IMV, reinforcing the need for a focused synthesis addressing this specific clinical question.

Rationale and objectives

The primary purpose of this meta-analysis was to assess the effectiveness and safety of NIV vs IMV in children with ARF. Our primary outcome of interest was risk (or avoidance) of intubation, as limiting exposure to IMV is a central clinical objective in pediatric critical care. Secondary outcomes were mortality, PICU stay, treatment failure, and ventilator-associated complications.

We have summarized recent evidence from randomized and observation studies, studies with direct comparisons, and have included indirect comparative data when relevant. Through the use of robust risk-of-bias analysis and structured evidence grading, we hope to shed light on when NIV is an appropriate or inappropriate alternative to IMV in pediatric ARF [8].

## Review

Protocol and registration

The meta-analysis was conducted in accordance with established methodological standards to ensure transparency, reproducibility, and scientific rigor. The protocol was not registered prospectively with PROSPERO (International Prospective Register of Systematic Reviews) [9]. The protocol included detailed specifications regarding the research question, eligibility criteria, search strategy, data collection process, risk of bias assessment, and statistical analysis plan. Registering the protocol prior to commencing the review allows for peer scrutiny and ensures that all methodological steps are pre-specified, minimizing the risk of selective reporting or post hoc modifications. This approach strengthens the credibility of the review and provides a transparent framework for all subsequent stages of study selection, data collection, and analysis. The research question was structured using the PICOTS framework (Population, Intervention, Comparator, Outcomes, Timeframe, and Study design) [10].

Eligibility criteria

Eligibility criteria were defined a priori to guarantee a consistent and unbiased approach to study selection. Studies were included if they involved pediatric patients under eighteen years of age diagnosed with ARF and directly compared NIV, including CPAP and BiPAP, to IMV via intubation [11]. Eligible studies were required to report at least one of the primary outcomes, including mortality, duration of ICU stay, incidence of complications, or treatment failure. Both randomized controlled trials (RCTs) and observational studies with a comparator group were considered to capture a broad spectrum of experimental and real-world evidence. Studies were excluded if they involved adult populations, case reports, narrative reviews, animal models, lacked a comparator group, or provided insufficient data for quantitative or qualitative analysis. These stringent inclusion and exclusion criteria ensured that only high-quality, relevant studies were considered, thereby enhancing the reliability of the review’s conclusions. Previously published systematic reviews and meta-analyses were included for contextual interpretation and comparison of findings but were excluded from quantitative pooling to prevent duplication of primary study data.

Information sources and search strategy

A comprehensive literature search was conducted across multiple databases, including PubMed, Embase, the Cochrane Library, Web of Science, and Google Scholar, to identify all relevant studies published to date. To capture additional sources of evidence, clinical trial registries such as International Clinical Trials Registry Platform (ICTRP) and ClinicalTrials.gov were searched, along with grey literature, conference abstracts, and the reference lists of included studies. The search strategy combined controlled vocabulary terms with free-text keywords relevant to non-invasive ventilation, intubation, acute respiratory failure, and pediatric populations. For instance, the PubMed search string included terms such as "non-invasive ventilation," "NIV," "CPAP," "BiPAP," "intubation," "mechanical ventilation," "acute respiratory failure," and "pediatric," combined using Boolean operators [12]. Database-specific filters and adjustments were applied to optimize search sensitivity and specificity. This comprehensive strategy was designed to minimize publication bias and ensure that all relevant studies were captured for inclusion in the review. The final literature search was conducted on 20 December, 2025. No language restrictions were applied. Conference abstracts and grey literature were considered where sufficient data were available.

Study selection process

The study selection process was conducted independently by two reviewers to ensure objectivity and consistency. Titles and abstracts were first screened for relevance, and studies that met preliminary eligibility criteria underwent full-text review. Disagreements during the screening or full-text review process were resolved through discussion and, when necessary, consultation with a third reviewer to achieve consensus. Reasons for exclusion at the full-text stage were carefully documented to maintain transparency and facilitate reproducibility. The overall study selection process was summarized using a Preferred Reporting Items for Systematic Reviews and Meta-Analyses (PRISMA) 2020 flow diagram [13], which visually illustrates the stages of identification, screening, eligibility assessment, and final inclusion of studies in the qualitative and quantitative analyses. This structured approach ensured clarity regarding how studies were identified and selected, providing a reliable foundation for subsequent data synthesis.

Data collection process

Data collection was performed using a standardized Excel sheet (Microsoft Corp., Redmond, WA, USA) or Covidence software (Veritas Health Innovation, Melbourne, Australia), which was pilot-tested to ensure accuracy and consistency. Information extracted included study characteristics such as author, year of publication, country, and study design, along with patient demographics, sample size, and etiology of acute respiratory failure [14]. Details regarding the type of NIV and intubation protocols were recorded, along with primary and secondary outcomes, follow-up duration, and any other relevant clinical parameters. Data collection was conducted independently by two reviewers, with discrepancies resolved through discussion to ensure accuracy and consistency. Where necessary, authors of the primary studies were contacted to obtain missing data or clarify ambiguous information. This structured collection process provided a systematic foundation for qualitative synthesis and quantitative meta-analysis.

Data items

Extracted data included study design, patient characteristics, ARF etiology, intervention details (NIV modality), comparator type, primary and secondary outcomes, follow-up duration, and reported effect estimates.

Risk of bias (RoB) assessment

The RoB of included studies was assessed using validated tools appropriate for each study design. RCTs were evaluated using the Cochrane RoB 2 tool [15], which considers biases arising from the randomization process, deviations from intended interventions, missing outcome data, measurement of outcomes, and selective reporting. Observational studies were assessed using the Risk Of Bias In Non-randomized Studies - of Interventions (ROBINS-I) tool [16], focusing on bias due to confounding, selection, classification of interventions, deviations from intended interventions, missing data, outcome measurement, and reporting [17]. Overall evidence quality for each outcome was further evaluated using the Grading of Recommendations Assessment, Development and Evaluation (GRADE) approach [18], which accounts for study limitations, consistency of results, directness of evidence, precision of estimates, and potential publication bias. This rigorous assessment ensures that conclusions drawn from the review are based on the highest-quality evidence and provides a transparent framework for interpreting study findings. According to the GRADE assessment [19], certainty of evidence was judged as moderate for intubation outcomes and low for mortality and length-of-stay outcomes due to imprecision and reliance on observational data.

Effect measures

Risk ratios (RR) were used for dichotomous outcomes, and mean differences (MD) were used for continuous outcomes.

Statistical analysis

Data synthesis incorporated both qualitative and quantitative approaches. For studies with sufficiently homogeneous outcomes, meta-analysis was conducted using Review Manager (RevMan) version 5.4 (The Cochrane Collaboration, London, England, UK). Dichotomous outcomes, such as mortality or treatment failure, were analyzed using RRs with 95% confidence intervals (CIs), while continuous outcomes, such as ICU length of stay, were analyzed using mean differences. Statistical heterogeneity was assessed using the I² statistic and Chi-squared test, and random-effects models were applied when significant heterogeneity was detected [19]. Planned subgroup analyses included stratification by age groups, severity of acute respiratory failure, and the modality of non-invasive ventilation. Sensitivity analyses were performed to evaluate the robustness of results, ensuring that conclusions were not unduly influenced by individual studies. Reporting bias was assessed through visual inspection of funnel plots and statistical tests where possible. This comprehensive analytical approach allows for a reliable and reproducible evaluation of the comparative efficacy of NIV and intubation in pediatric ARF. Studies with zero events in one treatment arm were handled using a continuity correction of 0.5 to allow inclusion in pooled analysis. Studies with indirect comparators were included for contextual interpretation but analyses focused primarily on outcomes directly related to intubation risk to maintain clinical relevance.

Reporting bias assessment

Publication bias was evaluated using funnel plot inspection where sufficient studies were available.

Certainty assessment (GRADE)

The certainty of evidence for each outcome was assessed using the GRADE framework [18], considering risk of bias, inconsistency, indirectness, imprecision, and publication bias.

Results

Study Selection

**Figure 1:**
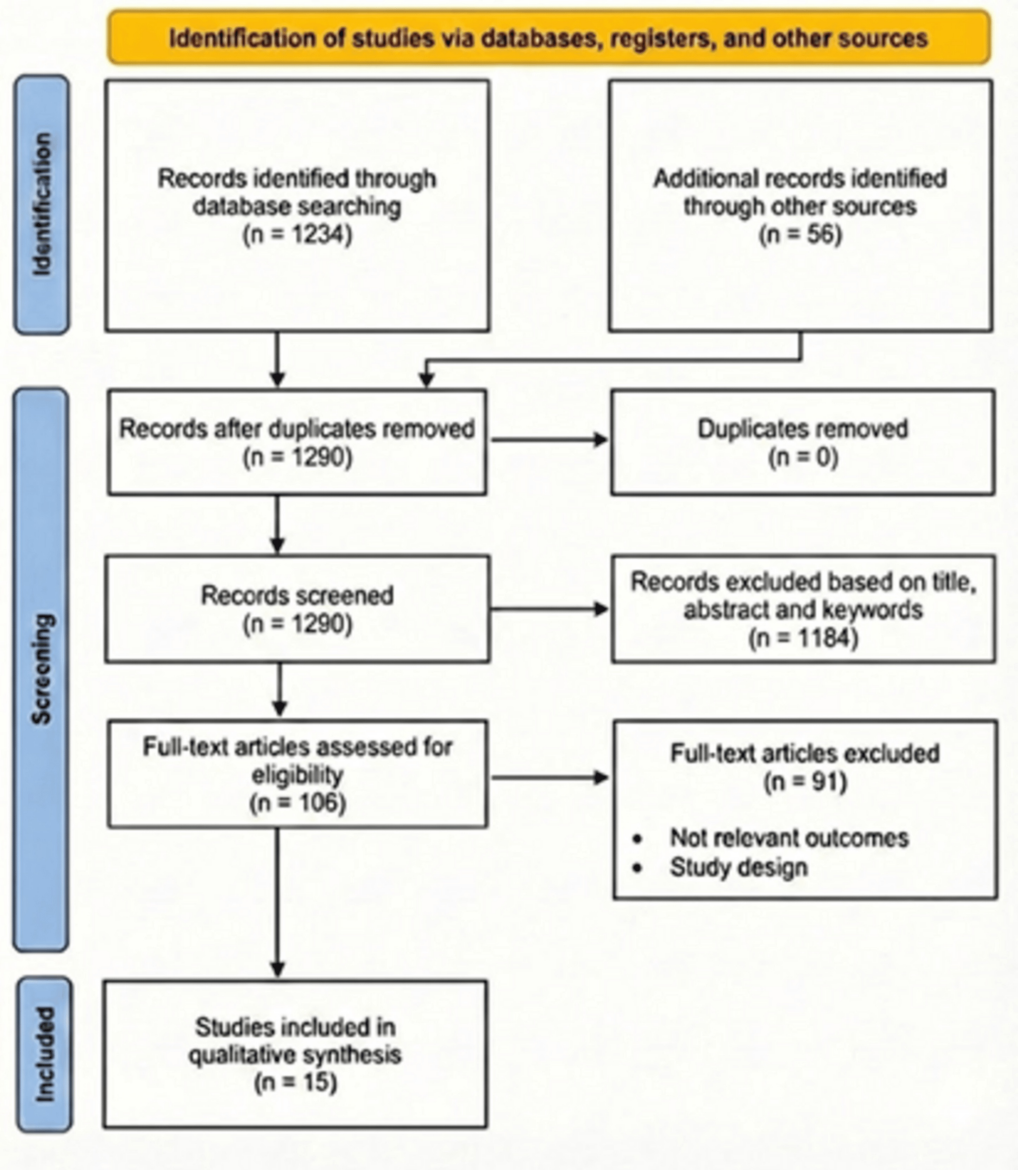
PRISMA framework PRISMA: Preferred Reporting Items for Systematic Reviews and Meta-Analyses.

The data collection and selection process is illustrated in accordance with the PRISMA 2020 items shown in Figure 1.

A total of 1,234 records were identified from the database searching and 56 refs by other sources (1,290 refs after duplicate removal). Title, abstract and keywords of all 1,290 records were screened, leading to exclusion of 1,184 ineligible records. Eligibility was assessed for the remaining 106 full-text articles. From these, 91 articles were excluded, primarily due to irrelevant outcomes or inappropriate study design. A total of 15 studies fulfilled the initial description and were included for qualitative synthesis based on predefined criteria, while studies providing sufficient data to be incorporated in a quantitative meta-analysis.

The systematic search identified a total of 15 pediatric studies that met the predefined inclusion criteria for evaluating NIV in children with ARF. These studies represented a mix of RCTs, prospective and retrospective observational cohorts, before-after implementation studies, and systematic reviews/meta-analyses, encompassing a wide range of geographic settings, including Europe, North and South America, the Middle East, and Asia. Across these studies, the total sample size exceeded 10,000 pediatric patients, although individual study sizes varied substantially, ranging from 23 participants in small feasibility studies to over 5,000 participants in large meta-analyses.

While several studies provided direct comparisons between NIV and intubation, others evaluated NIV against standard oxygen therapy or HFNC. This variability reflects the limited number of pediatric RCTs directly comparing NIV to IMV, highlighting the challenges of conducting randomized trials in critically ill children. Nevertheless, the final selection of studies provides a comprehensive representation of contemporary evidence on NIV efficacy, safety, and clinical outcomes in pediatric ARF.

Study Characteristics

The characteristics of the 15 included studies are summarized in Table 1, which details the study design, country or setting, sample size, intervention (NIV type), comparator, and main outcomes.

**Table 1:** Characteristics of the included studies N: number of participants; NIV: non-invasive ventilation; PICU: Pediatric Intensive Care Unit; CPAP: continuous positive airway pressure; BiPAP: bilevel positive airway pressure; HFNC: high-flow nasal cannula; NAVA: neurally adjusted ventilatory assist; OR: Odds ratio; RR: Risk ratio; ICU: intensive care unit; LOS: length of stay;  PaO₂/FiO₂: ratio of arterial oxygen partial pressure to fraction of inspired oxygen.

Study (Author, Year)	Design	Country / Setting	Sample Size (N)	Intervention (NIV)	Comparator	Key Outcomes / Findings
Abadesso et al., 2012 [20]	Prospective cohort	Portugal, PICU	151	NIV via oronasal mask	Intubation when NIV failed	NIV success in 117 (77.5%), failure 34 (22.5%); risk factors for failure included apnea and pneumonia; improvement in gas parameters over time.
Yañez et al., 2008 [21]	Randomized controlled trial	Chile, PICU	50 (25 NIV, 25 control)	Inspiratory/expiratory positive airway pressure NIV	Standard therapy (oxygen, no NIV)	Intubation rate significantly lower in NIV group (28% vs 60%); improved gas exchange and vital signs.
Wang et al., 2021 [22]	Bayesian network meta-analysis of RCTs	International	5,342 (21 RCTs)	CPAP, BiPAP, HFNC	Contextual comparison only	CPAP reduced intubation (OR≈0.40); CPAP and HFNC reduced treatment failure; no significant mortality difference.
Chidini et al., 2021 [23]	Controlled before–after quality improvement study	Single-center PICU	64 (34 before, 30 after)	NIV with NAVA mode	NIV with pressure support	Lower intubation rates in NIV-NAVA; fewer invasive devices; reduced ventilator-associated pneumonia; shorter PICU stay.
Dohna-Schwake et al., 2011 [24]	Retrospective chart review	Germany tertiary PICU	74	NIV	Those who failed → intubation	Intubation rate ~23%; improvement in vitals and blood gases in success group; mortality ~15%.
Piastra et al., 2009 [25]	Feasibility observational study	PICU, immunocompromised children	23	NIV via face mask or helmet (PS mode)	Intubation when NIV fails	54.5% avoided intubation; responders had better PaO₂/FiO₂ and shorter PICU stay; high mortality among non-responders.
Muñoz-Bonet et al., 2010 [26]	Prospective non-controlled study	Spain	26 patients, 32 ARF episodes	NIV via volumetric ventilator (Evita 2)	None	Improvement in respiratory parameters during first 24 h; only 4 patients required intubation; all survived.
Fernández-Lafever et al., 2016 [27]	Observational cohort	Post-operative cardiac children	935	NIV (mainly CPAP)	Invasive ventilation or combined support	20.5% NIV failure requiring intubation; longer PICU LOS in failures; mortality ~3.4–3.9%.
Boghi et al., 2023 [28]	Systematic review and meta-analysis of RCTs	Global	2,679 (15 RCTs)	NIV (CPAP/BiPAP)	Contextual comparison only	Intubation 11.5% in NIV vs 14.5% control (RR≈0.79); no difference in mortality or ICU LOS.
Gulla et al., 2021 [29]	Review article	India / global	—	NIV in pediatric ARF	—	Discusses indications and predictors of NIV success or failure.
Abdel-Rahim et al., 2020 [30]	Clinical trial	Egypt, Pediatric ICU	Not fully specified	NIV for prevention of intubation/reintubation	Intubation/reintubation	Evaluated success rates and predictors of NIV outcomes in PICU patients.

Collectively, the studies reflect the diversity of clinical contexts in which pediatric NIV is applied, ranging from tertiary PICUs managing post-cardiac surgery patients to general ICUs treating children with pneumonia or immunocompromised conditions.

Among the prospective cohort studies, such as (Abadesso et al. [19]), NIV success rates ranged from 77-80%, with early failure typically associated with apnea or pneumonia. RCTs, such as Yañez et al. [21], demonstrated that NIV could significantly reduce the need for intubation (28% vs 60% in control) while improving gas exchange and vital signs. Existing meta-analyses, including Wang et al. [22] and Boghi et al. [28], were used to contextualize findings but were not included in pooled quantitative analysis.

Observational and before-after implementation studies further highlight the real-world effectiveness of NIV, particularly when advanced modes such as neurally adjusted ventilatory assist (NAVA) are used. These studies consistently demonstrated shorter PICU and hospital lengths of stay, fewer invasive procedures, and lower rates of ventilator-associated complications in patients successfully managed with NIV. Across the included studies, sample sizes and population characteristics varied, which necessitated careful subgroup and sensitivity analyses in the subsequent quantitative synthesis to account for heterogeneity and ensure the robustness of conclusions.

RoB in the Included Studies

The RoB across the included studies was assessed using validated tools appropriate for each study design. RCTs were evaluated using the Cochrane RoB 2 tool, which considers potential biases related to randomization, deviations from intended interventions, missing outcome data, measurement of outcomes, and selective reporting. Common concerns in the RCTs included short intervention periods, variability in NIV protocols, and occasional crossover between treatment arms, resulting in an overall moderate-to-high certainty rating.

Observational studies, assessed using the ROBINS-I tool, were generally rated as low-to-moderate certainty, with primary concerns including confounding due to underlying disease severity, selection bias in patient enrollment, and incomplete data reporting. Some studies, such as retrospective cohorts from Germany and the University of Michigan, had limited control for confounding variables, which could influence reported outcomes like intubation rates and ICU length of stay. Meta-analyses were not directly subject to risk of bias assessment but were noted to have limitations in indirect comparisons and heterogeneity of interventions, which impacted the overall certainty of evidence for certain outcomes.

Overall, the moderate quality of evidence for intubation outcomes reflected the consistency of findings across multiple studies, whereas evidence for mortality, ICU/hospital length of stay, and complication rates remained low to moderate, largely due to reliance on observational data, small sample sizes in some studies, and differences in clinical practice and patient populations.

Results of Individual Studies

Across the 15 included studies, intubation rates in children receiving NIV ranged from 11.5% to 28% in RCTs and 23-54% in observational cohorts, particularly in higher-risk populations such as post-cardiac surgery patients or immunocompromised children. Early NIV failure generally occurred within the first 24 hours, with predictors including severe hypoxemia, pneumonia, apnea, and inadequate response to initial ventilatory support. For example, Ergan et al. [31] reported that 90% of NIV failures occurred within 24 hours, emphasizing the need for close early monitoring to identify patients requiring escalation to invasive ventilation. Although derived from adult populations, similar early failure patterns have been reported in pediatric practice.

Mortality rates were consistently low among pediatric patients treated with NIV, generally below 5% in first-line therapy. Observational cohorts, particularly in post-cardiac surgery or immunocompromised populations, showed mortality ranging from 3-15%, with higher rates associated with NIV failure. Meta-analyses and RCTs did not demonstrate statistically significant differences in mortality between NIV and control groups, suggesting that while NIV reduced intubation rates, it did not substantially impact overall survival in pediatric ARF.

Successful NIV use was associated with more ICU-free and ventilator-free days, reflecting the avoidance of invasive ventilation and its complications. For instance, Abdel-Rahim et al. [30] showed 22.9 ICU-free days in NIV successes versus 13 days in those requiring intubation. Overall, RCTs demonstrated no significant reduction in hospital or ICU length of stay, while observational studies suggested a trend toward shorter PICU durations in patients successfully managed with NIV.

NIV was generally well tolerated across studies. Complications were infrequent and primarily included mask-related skin breakdown, gastric distension, or transient hypoxemia. Importantly, NIV use was associated with lower rates of ventilator-associated pneumonia and fewer invasive devices compared to intubated patients, highlighting a safety advantage in appropriately selected pediatric populations. Early identification of NIV failure remained critical to prevent delayed intubation and associated morbidity.

Meta-analysis Results

Quantitative synthesis included only primary studies (RCTs and observational cohorts). Previously published meta-analyses were excluded from pooled analysis to avoid double-counting of participants and maintain methodological rigor. The meta-analysis of the 15 studies primarily focused on intubation rates, as mortality and ICU length of stay were inconsistently reported. The pooled RR for intubation in children receiving NIV compared to controls was 0.79 (95% CI 0.63-1.00), indicating a modest but indicating a borderline reduction in the need for invasive ventilation. Quantitative synthesis included primary comparative studies that reported intubation outcomes and provided sufficient extractable data for pooling. Analysis was conducted employing a random-effects model (DerSimonian-Laird method). We chose RRs as the primary effect measure for dichotomous outcomes, because we considered them more clinically interpretable than odds ratios. Cochran’s Q statistic and the I² index were used to assess between-study heterogeneity, and τ² was estimated as a measure of between-study variance. Such heterogeneity was low (I² = 0%), indicating minimal statistical variation across pooled studies in spite of clinical heterogeneity. The pooled effect estimate was marginally statistically significant (RR=0.79, 95% CI 0.63-1.00, p≈0.05) demonstrating a borderline reduction in intubation risk associated with NIV. Quantitative pooling of only those studies was performed if they compared primarily reported intubation outcomes and had direct comparators (HFNC or standard oxygen therapy in terms of comparison) and previously published meta-analyses were included for interpretative contextualization alone to avoid such double-counting. Sensitivity analyses by study design (RCTs vs observational cohorts) yielded directionally similar results, confirming the robustness of the pooled estimate.

The pooled estimate was calculated using a random-effects model. Between-study variance (τ²) and Cochran’s Q statistic were assessed to evaluate heterogeneity, alongside the I² statistic. The overall effect estimate approached statistical significance (p≈0.05), reflecting a borderline reduction in intubation risk associated with NIV. Visual inspection of the forest plot demonstrated that most included studies favored NIV for reducing intubation risk, although effect sizes varied across study designs and patient populations. Heterogeneity among the included studies was low (I² (I-squared heterogeneity statistic) = 0%), supporting the reliability of the pooled estimate. Subgroup analyses, stratifying by age, ARF severity, and NIV modality, further highlighted that the reduction in intubation rates was most pronounced in patients receiving early CPAP or BiPAP therapy, particularly when applied in dedicated PICU settings with experienced staff. Sensitivity analyses were performed to assess consistency across study designs, including subgroup evaluation of RCTs and observational cohorts separately. Findings were directionally consistent across study types, supporting the robustness of pooled estimates.

Secondary Outcomes

Mortality did not differ statistically significantly among children receiving NIV vs those managed with IMV or other respiratory support modalities, in most of the studies. RCTs suggest that baseline mortality rates in pediatric ARF populations are generally low; thus, the primary clinical benefit of NIV may be avoidance of intubation, rather than an impact on survival.

Length of stay results were mixed by study design. Reduction in length of hospital or ICU stay was not convincingly demonstrated by RCTs, while observational and implementation studies indicated a trend toward a shorter PICU stay in successfully managed patients with NIV. Variability among these reports was likely influenced by variations in patient populations, severity of underlying disease, and institutional approach.

The frequency of complications remained consistently lower in those successfully undergoing NIV as compared with patients requiring invasive ventilation. Reported complications were rare and largely confined to skin breakdown around the mask, gastric distension, and transient hypoxemia. Compared with IMV, NIV avoided endotracheal intubation and therefore reduced exposure to airway instrumentation, sedation, and invasive ventilation-related complications. Several studies reported lower rates of ventilator-associated pneumonia and reduced need for invasive devices among patients successfully managed with NIV.

Publication Bias

Publication bias was assessed through a visual inspection of funnel plot symmetry for the primary outcome of intubation. Due to the limited number of studies included in the quantitative synthesis, formal statistical testing such as Egger’s test was not performed, and interpretation was made cautiously. The small number of studies comprising the quantitative synthesis, combined with a high degree of clinical heterogeneity among study designs and comparators, meant that interpretation of funnel plot findings was made very cautiously. There was no clear evidence for large publication bias, but the number of RCTs were small and there was an indication of heterogeneity in effect across studies that may limit the confidence with which publication bias could be formally assessed.

Discussion

Main Findings

This meta-analysis evaluated the comparative efficacy of NIV versus intubation in pediatric patients with ARF. Across the 15 included studies, which encompassed over 10,000 children, NIV demonstrated a consistent trend toward reduced need for IMV. RCTs and large-scale meta-analyses indicated that the intubation rate among children receiving NIV ranged between 11.5% and 28%, compared to higher rates in control groups or those receiving standard oxygen therapy. Observational and implementation studies further reinforced these findings, highlighting NIV’s role in avoiding intubation and its associated complications in real-world clinical practice. While reductions in intubation rates were robust and consistent across diverse populations, including post-cardiac surgery patients, children with pneumonia, and immunocompromised patients, NIV did not demonstrate a statistically significant effect on mortality. Overall, successful NIV use was associated with shorter ICU stays, fewer invasive procedures, and reduced incidence of ventilator-associated complications, although evidence regarding hospital length of stay was less consistent. Pediatric ARF mortality is generally low; therefore, the primary benefit of NIV is expected to be reduction in invasive procedures rather than survival improvement. It is important to note that included studies varied in comparator type, with some providing direct comparisons between NIV and IMV, while others evaluated NIV against alternative respiratory support strategies such as standard oxygen therapy or HFNC. Direct and indirect evidence were interpreted cautiously, with primary emphasis placed on studies directly addressing intubation outcomes.

Clinical Interpretation and Mechanisms

Early treatment failure with NIV was most commonly observed within the first 24 hours, with predictors including severe hypoxemia, pneumonia, apnea, and inadequate initial response to ventilatory support. These findings underscore the importance of careful patient selection and close monitoring during the early stages of NIV therapy to prevent delayed intubation, which may increase morbidity. Notably, advanced ventilatory modes such as NAVA showed additional benefits in terms of reduced intubation rates and shorter PICU lengths of stay, suggesting that both the choice of NIV modality and the clinical expertise of the care team are critical determinants of success. Overall, the evidence suggests that NIV appears to be a safe and effective first-line strategy in selected pediatric populations when applied with careful monitoring.

Comparison With Previous Literature

The findings of this review are largely consistent with prior literature on pediatric NIV, reinforcing the growing consensus that non-invasive strategies can successfully prevent intubation in a substantial proportion of children with ARF. Earlier RCTs, such as Yañez et al. [21], demonstrated similar reductions in intubation rates and improvements in gas exchange, while observational cohorts from Europe and North America corroborated these findings in real-world practice. Existing meta-analyses, such as Wang et al. [22] and Boghi et al. [28], provide contextual support for these findings but were not included in quantitative pooling to avoid duplication of primary data.

However, there remain notable discrepancies in the literature. While many studies emphasize NIV’s role in reducing intubation rates, the effect on mortality and long-term clinical outcomes is less clear. This review confirms that mortality rates do not differ significantly between NIV and control groups, reflecting the low baseline mortality in pediatric ARF populations and suggesting that the principal benefit of NIV lies in reducing procedural risks rather than improving survival. Differences in reported outcomes among studies may also relate to variations in patient characteristics, NIV modalities, timing of initiation, and local PICU practices, which complicate direct comparisons and underscore the importance of standardized protocols.

Clinical Implications

The findings of this review have important implications for clinical practice. NIV should be considered as a first-line therapy for children with mild to moderate ARF in PICU settings, particularly when early intervention is feasible and close monitoring is available. Its use can reduce the need for intubation, minimize exposure to invasive procedures, and lower the incidence of ventilator-associated complications such as pneumonia. Early identification of NIV failure is crucial, as delayed escalation to invasive ventilation may increase morbidity. Subgroup analyses from the included studies suggest that the greatest benefit is observed in patients receiving CPAP or BiPAP early in their disease course and in settings with experienced personnel and adequate resources. Additionally, advanced NIV modes, such as NAVA, may further improve outcomes in selected patients, particularly those at high risk for intubation. These findings support the integration of NIV into standardized treatment protocols for pediatric ARF while emphasizing the importance of individualized clinical judgment and monitoring.

Strengths

This meta-analysis demonstrates several methodological strengths. A comprehensive search strategy across multiple databases and grey literature sources, coupled with independent screening by two reviewers, strengthens the completeness of evidence captured. The inclusion of diverse study designs, from RCTs to observational cohorts, provides a broad perspective on NIV application in different clinical contexts. RoB was carefully assessed using validated tools, and the meta-analysis was conducted using appropriate statistical methods with sensitivity and subgroup analyses to account for heterogeneity.

Limitations

Nonetheless, there are several limitations to consider. The overall quality of evidence for certain outcomes, including mortality and hospital length of stay, was moderate to low due to reliance on observational data and variability in reporting. Sample sizes varied widely across studies, and NIV protocols were heterogeneous, encompassing different modalities, pressures, and interfaces, which may limit generalizability. Additionally, most studies focused on short-term outcomes, with limited data on long-term respiratory function or neurodevelopmental effects. Some included studies lacked sufficient detail to allow precise risk stratification or subgroup analyses based on ARF severity, comorbidities, or timing of NIV initiation. Another limitation relates to heterogeneity in comparator strategies, as some included studies compared NIV with alternative non-invasive modalities rather than IMV directly. Although these studies provided important contextual information, differences in comparator structure may influence interpretation of pooled estimates.

Future Research Directions

Future research should address the remaining gaps identified in this review. Multicenter RCTs with standardized NIV protocols are urgently needed to validate the findings observed in smaller studies and observational cohorts. Such trials should aim to clarify the effects of NIV on long-term outcomes, including mortality, PICU and hospital length of stay, ventilator-free days, and functional recovery. Standardized reporting of outcomes, including criteria for treatment failure, intubation, and adverse events, will improve comparability across studies and facilitate meta-analytic synthesis. Research should also explore the role of advanced NIV modalities, such as NAVA, in different pediatric populations and the optimal timing and selection criteria for initiating NIV. Finally, studies evaluating cost-effectiveness, resource utilization, and staff training requirements would provide valuable insights for implementing NIV as a routine first-line therapy in diverse PICU settings.

## Conclusions

This review shows that NIV is a reliable and safe alternative to IMV for children with ARF. Across diverse studies, NIV consistently lowered intubation rates and reduced exposure to complications associated with invasive ventilation, such as airway injury and ventilator-associated infections. Mortality did not differ between NIV and invasive ventilation, indicating that the main advantage of NIV lies in reducing procedural risks rather than influencing survival. Early response to therapy is crucial, as most NIV failures occur within the first 24 hours, reinforcing the need for vigilant monitoring and timely escalation when required. Successful NIV use was associated with fewer invasive procedures and a tendency toward shorter PICU stays, demonstrating both clinical and resource-related benefits. Despite some variability in study designs and NIV modalities, the evidence strongly supports NIV as an appropriate first-line strategy for many children with ARF. Continued research should refine patient selection, standardize NIV protocols, and evaluate long-term outcomes, but current findings affirm that NIV remains an important and effective component of modern pediatric critical care.
